# Alternative splicing of estrogen receptor alpha in hepatocellular carcinoma

**DOI:** 10.1186/s12885-016-2928-3

**Published:** 2016-11-30

**Authors:** Jian Zhang, Jianwei Ren, Jiamin Wei, Charing C. N. Chong, Dongjie Yang, Yulong He, George G. Chen, Paul B. S. Lai

**Affiliations:** 1 Department of Surgery, The Chinese University of Hong Kong, Prince of Wales Hospital, Shatin, New Territories, Hong Kong, Special Administrative Region of China; 2 Shenzhen Research Institute, the Chinese University of Hong Kong, Shenzhen, China; 3 Division of Gastrointestinal Surgery & Gastric Cancer Center, The First Affiliated Hospital of Sun Yat-sen University, Guangzhou, China; 4 The Third Affiliated Hospital of Sun Yat-sen University, Guangzhou, China

**Keywords:** Hepatocellular carcinoma, Estrogen receptors, HCC survival

## Abstract

**Background:**

The role of estrogen receptor alpha (ERa), estrogen receptor beta (ERb) and ERa36 signaling in hepatocellular carcinoma (HCC) is not fully addressed.

**Methods:**

In this study, three cohorts were included: (i) primary HCC patients (*N* = 76, cohort P), (ii) colorectal liver metastasis (mCRC) (*N* = 32, cohort S), and (iii) HCC from The Cancer Genome Atlas (TCGA) (*N* = 121). The levels of ERa36 and wtER36 were measured and their correlation with clinicopathologic features was determined.

**Results:**

WtERa was downregulated and that ERa36 was upregulated in tumor tissues in both cohort P and TCGA data set. ERa36 was downregulated in tumor tissues in cohort S. In cohort P, wtERa was differentially expressed in gender (*P* < 0.000), age (*P* = 0.004), tumor number (*P* = 0.043), tumor size (*P* = 0.002), intrahepatic recurrence (*P* = 0.054). ERa36 was unequally expressed in different non-tumor liver status (*P* = 0.040). WtERa was negatively associated with overall survival (OS) and disease free survival (DFS) in cohort P. Compared with non-tumor tissues, the expression of ERa36 was increased in primary HCC but decreased in secondary HCC, showing opposite expression patterns of ERa36 between primary HCC and secondary HCC.

**Conclusions:**

Primary HCC is associated with the decreased WtERa but increased ERa36. The expression pattern of ERa36 is different between primary HCC and secondary HCC, as the former with the increased ERa36 but the latter with the decreased ERa36. Therefore, the expression of ERa36 may be used to differentiate the primary HCC and the secondary one.

## Background

Despite decades of research, the etiology of HCC remains unclear. Hepatocellular carcinoma (HCC) is a common cancer and leading cause of cancer death worldwide [1]. While rates of new cases and deaths have fallen steadily in recent years, these declines have been slow in magnitude compared with other cancers. Therefore, the prevention and treatment of HCC remain unmet needs. The identification of key biomarkers with prognostic value would help guide future clinical trials of HCC therapies.

Estrogens play an important role in normal liver function as well as HCC progression [2]. The classical two receptors of estrogen are estrogen receptor alpha (ERa) and beta (ERb), and ERa is the dominant ER receptor in hepatocytes [3]. ERα and its’ variants are expressed in HCC, and the presence of ERa has been regarded as an indicator for anti-hormonal (tamoxifen) therapy [4, 5]. However, tamoxifen did not improve the survival or quality of life in advanced stage HCC patients [6, 7] and further clinical development was stopped. The presence of ER transcripts in inoperable HCC is a strong negative predictor of survival [8].

Alternative splicing has critical roles in normal development and can promote growth and survival in cancer [9]. Due to alternative RNA splicing, several ER isoforms are formed. So far, at least three ERa isoforms have been identified, and their molecular weights are 66, 46 and 36 kDa respectively [10]. Compared with ERa66 or ERa46, ER-a36 is different in its structure, expression pattern and function [11].

In this study, we analyzed the expression of wtERa and ERa36 in HCC biopsy samples using two cohorts of tissues annotated for relevant histological and clinical variables. We then assessed the relationship of these markers with patients’ survival. In addition, alterations in ER mRNA and protein expression and their relationships with overall survival (OS) and disease-free survival (DFS) were evaluated using data from TCGA.

## Methods

### Patients and samples

Tumor samples were collected from 108 HCC patients who underwent surgery at the Prince of Wales Hospital, Hong Kong during 2000 to 2014. All patients were tested positive for HBsAg and negative for antibodies to the hepatitis C virus (anti-HCV) and human immunodeficiency virus (anti-HIV). All tumor tissues were histologically diagnosed and the stages at diagnosis were classified according to the criteria of the American Joint Committee on Cancer criteria. Tumor samples were divided into two cohorts: cohort P (*n* = 76), primary HCC patients, and cohort S (*n* = 32), colorectal liver metastasis (mCRC). All patients were successfully followed-up for 1203.5 ± 128.8 days (mean ± SD). All subjects provided their written informed consent prior to specimen collection. The study was carried out with the approval of the Joint CUHK-NTEC Clinical Research Ethics committee. The characteristics of patients were presented in Table 1. Detailed clinical and pathological information were available for most of these cases, including patients’ demographic data, pathologic tumor-node-metastasis (TNM) staging, OS time, and disease-free survival (DFS) time. Gender was well balanced in both of the cohorts.

**Table 1:** Patients’ characteristics in primary HCC (cohort P) and secondary HCC (cohort S) from mCRC

Clinical features		Cohort P (*n* = 76)	Cohort S (*n* = 32)	*P* value
Age (y)	≤58	32	13	0.887
	>58	44	19	
Gender	Female	39	14	0.473
	Male	37	18	
Liver cirrhosis	No	26	31	**0.000**
	Yes	50	1	
HBsAg	Negative	17	30	**0.000**
	Positive	59	2	
Preoperative liver function (Child-Pugh)	A	73	32	0.553
	NA	3	0	
AFP	≤18 ng/ml	33	30	**0.000**
	>18 ng/ml	42	2	
Tumor size	≤5 cm	51	26	0.138
	>5 cm	25	6	
Tumor number	Single	61	20	0.052
	Multiple	15	12	
Vascular invasion	No	63	29	0.384
	Yes	13	3	
Pathological Grade	Well	10	0	NA
	Moderate	58	1	
	Poor	8	1	
	NA	0	30	
Neo-adjuvant treatments before operation	No	72	28	0.233
	Yes	4	4	
Adjuvant treatments after operation	Yes	18	18	**0.001**
	No	58	14	
Intra-hepatic recurrence	No	44	29	**0.001**
	Yes	32	3	
Extra-hepatic metastasis	No	63	26	0.838
	Yes	13	6	
AJCC Stage	1	52	NA	NA
	2	16	NA	
	3 + 3A	8	NA	

*Abbreviations*: *AFP* alpha-fetoprotein, *AJCC* American Joint Committee on Cancer, *NA* not available; Neo-adjuvant treatments before operation, including chemotherapy, preoperative portal vein embolization (PVE) and transcatheter arterial chemoembolization (TACE); Adjuvant treatments after operation, including lipiodol-iodine-131, chemotherapy, TACE

Significant data are highlighted in bold

### Immunohistochemistry and analysis

The expression of wtERa was determined by immunohistochemical assay on paraffin-embedded tissue sections (5 μm). Sections were deparaffinized in xylene and dehydrated in series of graded ethanol. Antigen retrieval was carried out in microwave oven with 10 mM sodium citrate buffer (pH 6.0). The endogenous peroxidase activity was inhibited by incubating the tissues with 3 % H_2_O_2_ in TBS for 5 min and nonspecific binding sites were blocked by incubating with 5 % normal horse serum for another 30 min. The sections were then incubated in a humidified chamber overnight at 4 °C with primary rabbit antibody wtERα (Santa Cruz, SC-130072), ERa36 (Abgent, AP19657b), or ERb (Santa Cruz, SC-53494). The primary antibody was then rinsed by tris-buffered saline (TBS) at pH 7.4. Labeling was carried out with Vector Rabbit ImmPress HRP micropolymer for 30 min at room temperature. Target antigens were visualized with Vector ImmPACT DAB EqV Peroxidase (HRP) substrate.

Slides were counterstained with hematoxylin and prepared for evaluation. The results were examined by two pathologists independently. Cytoplasmic and/or membranous expression intensity and density were semi-quantified using a six-value score (0 to 5) as follows: no staining or staining observed in <30 % of tumor cells was scored as 0 or 1+; Very weak positive or weak positive staining in ⩾30 % and <60 % of tumor cells was scored as 2+ and 3+; and positive or strong positive staining in ⩾60 % of tumor cells was scored as 4+ and 5+, respectively. A tumor sample was considered positive if the score was above the median value of all samples and negative otherwise.

### Analysis of ERa alternative splicing from TCGA

The alternative splicing of ERa was generated from RNA-seq data which were acquired from TCGA data portal by TCGA SpliceSeq database [12]. Based on the coverage of different splicing isoforms, each alternative splicing event was assigned with a PSI (Percent Spliced In) value ranging from 0 to 1. To increase the read coverage, we filtered out low coverage samples with less than 500 reads. Independent *t* test was used to compare the difference between tumor and adjacent non-tumor tissues. Correlations between ERa mRNA expression and alternative splicing events were calculated using the spearman rank correlation, ρ (rho). We obtained the overall survival data of LIHC patients from TCGA and computed their probability of survival using a Kaplan-Meier survival plot and the log-rank *P* values.

### Statistical analysis

The differences of clinical features between two cohorts were evaluated using Chi-square tests (2-sided). The summary statistics for the biomarker expression levels according to patients’ characteristics were computed. The Pearson/nonparametric correlation test and *t* test were used to compare biomarker expression among different subgroups defined by clinical features, such as gender, pathological stage and tumor size. The OS and DFS of each subgroup of patients were determined by the Kaplan–Meier method and compared using the log-rank test. Cox proportional hazard models were used for multivariate analyses. The expression of wtERa, ERa36, ERb, age, gender, AFP (ng/ml), tumor number, tumor size (cm), vascular invasion, intra-hepatic recurrence, extra-hepatic metastasis, neo-adjuvant treatments, adjuvant treatments, liver cirrhosis and HBsAg status were included in multivariate analyses. All statistical tests were two-sided, and *P* ≤ 0.05 was considered significant.

## Results

### Patient characteristics

All 108 patients enrolled in our study were pathologically diagnosed with HCC and/or colorectal cancer. The age for all patients was 60.57 ± 10.80 (mean ± SD) years (range: 31–80). Among the 108 participants, patients are divided into two cohorts. As summarized in Table 1, no significant difference was observed in the distribution of age (*P* = 0.887), gender (*P* = 0.473), Child-Pugh score (*P* = 0.553) and extra-hepatic metastasis (*P* = 0.838) between the two cohorts. The male: female ratio is 1:1.11 and 1:0.78 in two cohorts. In all 108 patients, in the neo-adjuvant treatments before operation, five cases received chemotherapy, one case received portal vein embolization (PVE) and two cases received transcatheter arterial chemoembolization (TACE); in adjuvant treatments after operation, 18 cases received chemotherapy, 16 cases received lipiodol-iodine-131, two cases received TACE only or combined with chemotherapy.

### Expression of wtERa, ERa36 and ER-beta in 76 primary HCC tissues

**Fig. 1:**
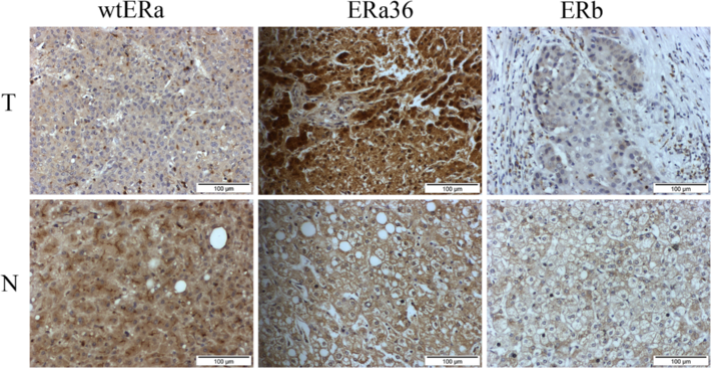
Representative photomicrographs of the immunohistochemical analysis of wtERa, ERa36 and ER-beta protein expression in 76 primary HCC (cohort P)

We performed an IHC analysis of wtERa, ERa36 and ER-beta expression in two HCC cohorts. Representative IHC results of cohort P (primary HCC) are shown in Fig. 1. Similar to the findings of breast cancer [13], wtERa expression was primarily in the cell membrane and cytoplasm of liver cells and significantly lower in HCC tissues than in adjacent non-tumor tissues (mean score 2.345 vs. 2.620, *P* < 0.010, Fig. 3). In contrast, ERa36 expression was primarily in cytoplasm of liver cells and significantly higher in HCC tissues than in adjacent non-tumor tissues (mean score 3.184 vs. 2.888, *P* < 0.008, Fig. 3). ERb expression was primarily in cytoplasm of liver cells and lower in HCC tissues than in adjacent non-tumor tissues (mean score 1.566 vs. 1.770, *P* = 0.139, Fig. 3).

### Expression of wtERa, ERa36 and ER-beta in 32 mCRC HCC tissues

**Fig. 2:**
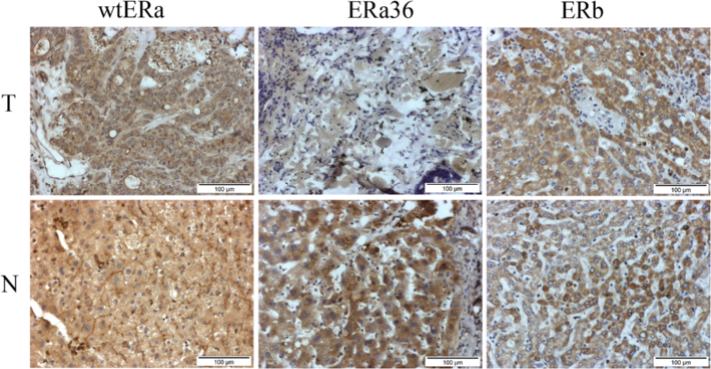
Representative photomicrographs of the immunohistochemical analysis of wtERa, ERa36 and ER-beta protein expression in 32 mCRC HCC tissues (cohort S)

**Fig. 3:**
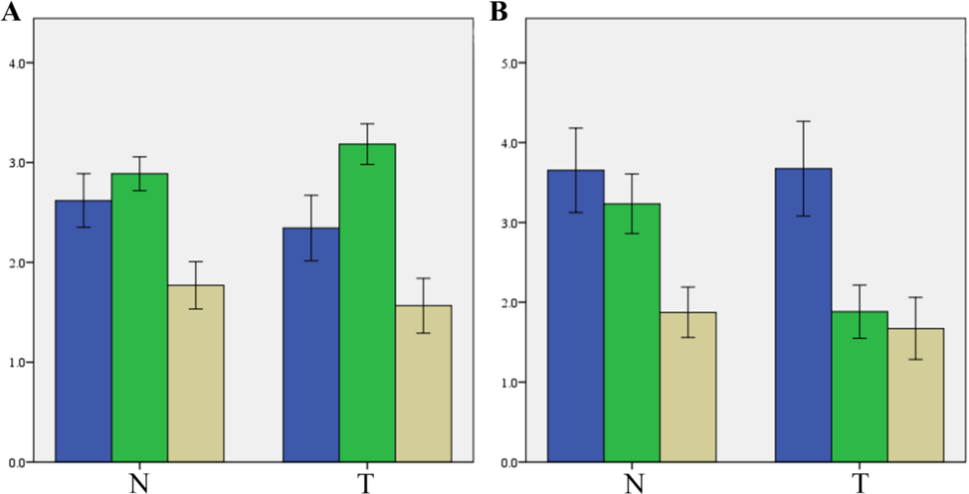
Mean expression scores of wtERa, ERa36 and ER-beta in primary HCC compared to secondary HCC. (**a**) Decreased wtERa (*P* < 0.010) and increased ERa36 (*P* < 0.008) in tumor tissues in Cohort P; and (**b**) Decreased ERa36 (*P* < 0.001) in tumor tissues in Cohort S. *P*-values were calculated using the paired sample *t* test. wtERa, *blue*; ERa36, *green*; ERb, *brown*; T, tumor tissue; N, adjacent non-tumor tissue; error bars, 95 % confident interval

Representative IHC results of cohort S (metastatic HCC) are shown in Fig. 2. It was found that the expression of either wtERa or ERb between mCRC HCC tissues and adjacent non-tumor tissues was not significantly different (*P* = 0.944 and *P* = 0.487) (Fig. 3). Interestingly, ERa36 expression was significantly lower in mCRC HCC tissues than in adjacent non-tumor tissues (mean score 1.883 vs. 3.234, *P* < 0.001) (Fig. 3). The expression of wtERa of both tumor and non-tumor tissues in cohort P was lower than in cohort S (*P* < 0.001). The expression of ERa36 in the tumor tissues of the cohort P was higher than in the cohort S (*P* < 0.001). There was no significant difference in ERb expression between the cohort P and the cohort S (Fig. 3).

### Correlation analysis of the expression of wtERa, ERa36 and ER-beta with clinical features

We further correlated the expression of wtERa, ERa36 and ERb with clinical features and patient survival. Using median expression values as cutoff points, the levels of wtERa, ERa36 and ERb were evaluated as ordinal variables (high expression vs. low expression).

**Fig. 4:**
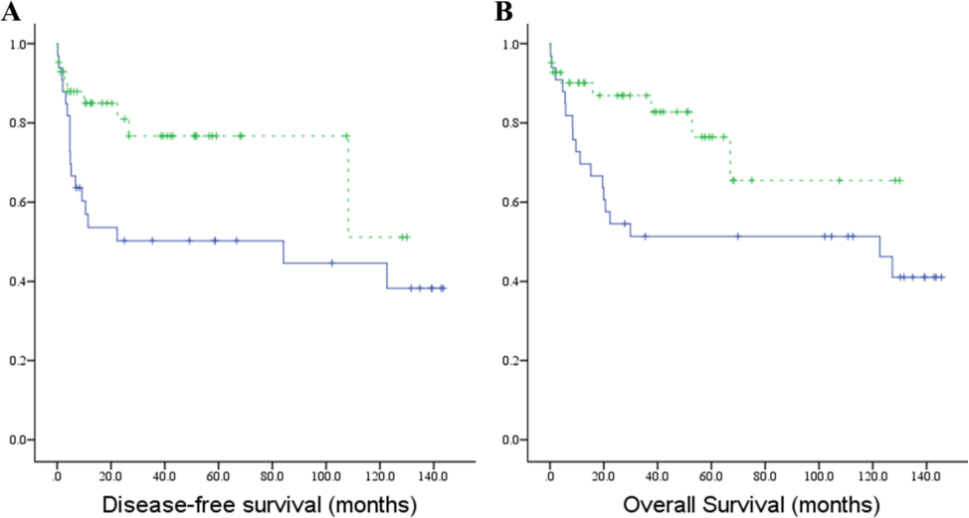
Kaplan-Meier survival curves for wtERa in cohort P. Using the median value as cutoff, cohort P was divided into two groups: each group with 38 cases. **a** Disease-free survival, **b** Overall survival (*Blue dotted line*, low expression; *Green dotted line*, high expression; Vertical axis, survival function)

In cohort P, wtERa was positively correlated with gender and age, but it was negatively correlated International Normalized Ratio (INR), size of the largest tumor, tumor recurrence or metastasis, and adjuvant treatments after operation (Table 2). ERa36 was positively correlated with the status of adjacent non-tumor liver tissues and liver cirrhosis, but it was negatively correlated with extra-hepatic metastasis and pathological grade. ERb was also positively correlated with gender and age, but it was negatively correlated with International Normalized Ratio (INR) and HBsAg status (Table 2). WtERa was negatively associated with OS and DFS in cohort P, while ERa36 and ERb were not associated with OS or DFS (Fig. 4). In cohort S, we did not find statistically significant changes in the expression of wtERa, or ERa36 or ERb under different clinical features, such as gender, age, vascular invasion, non-tumor liver status, HBsAg status, and survival.

**Table 2:** Correlation analysis of the expression of wtERa, ERa36 and ER-beta with clinical features (cohort P)

	Clinical features	Pearson correlation analysis	
		Correlation coefficient	*P* value^a^
wtERa	Gender (Female vs. Male)	0.662	0.000
	Age (>58 vs. ≤58)	0.276	0.007
	International Normalized Ratio	−0.442	0.000
	Size of the largest tumor (>5 cm vs. ≤5 cm)	−0.306	0.003
	Intra-hepatic recurrence (Yes vs. No)	−0.308	0.003
	Extra-hepatic metastasis (Yes vs. No)	−0.310	0.002
	Adjuvant treatments after operation (Yes vs. No)	−0.348	0.001
ERa36	Non-tumour liver (Cirrhosis vs. Fibrosis vs. Normal)	0.270	0.009
	Extra-hepatic metastasis (Yes vs. No)	−0.274	0.007
	Pathological Grade (Poor vs. Moderate vs. Well)	−0.274	0.007
	Liver cirrhosis (Yes vs. No)	0.267	0.009
ERb	Gender (Female vs. Male)	0.234	0.023
	Age (>58 vs. ≤58)	0.308	0.002
	International Normalized Ratio	−0.273	0.009
	HBsAg (+ vs. -)	−0.377	0.000

^a^ Correlation is significant at the 0.01 level (2-tailed)

The univariate analysis of the hazard ratios of clinical features and biomarkers for OS were summarized in Table 3 (cohort P). The result of the univariate analysis showed that among the clinical characteristics, gender, tumor number, tumor size, vascular invasion, extra-hepatic metastasis, neo-adjuvant treatments, adjuvant treatments, and liver cirrhosis were associated with patients’ OS, but wtERa, ERa36 and ERb were not (Table 3). In multivariate analysis, only gender, tumor size, adjuvant treatments, and liver cirrhosis appeared to be independent prognostic factors for OS and DFS prediction (*P* < 0.01, Table 3). Vascular invasion was also an independent prognostic factor for DFS (*P* = 0.037). WtERa, ERa36 and ERb levels were not significant markers for OS and DFS in the multivariate analysis (data not shown).

**Table 3:** Univariate and multivariate analyses of clinical features and biomarkers with patients’ overall survival (cohort P)

		Univariate analysis		Multivariate analysis	
		HR(95 % CI)	*P* value ^a^	HR(95 % CI)	*P* value^a^
wtERa	high vs. low	0.455 (0.202–1.025)	0.058	0.519 (0.156–1.723)	0.284
ERa36	high vs. low	1.070 (0.501–2.282)	0.862	2.853 (0.843–9.654)	0.092
ERb	high vs. low	1.121 (0.524–2.402)	0.768	1.151 (0.342–3.867)	0.820
Age	>58 vs. ≤58	1.536 (0.698–3.380)	0.286	1.767 (0.503–6.205)	0.375
Gender	Female vs. Male	0.314 (0.125–0.787)	**0.013**	0.186 (0.047–0.733)	**0.016**
AFP (ng/ml)	>18 vs. ≤18	0.896 (0.415–1.935)	0.780	0.978 (0.327–2.925)	0.969
Tumor number	Multiple vs. Single	4.660 (2.069–10.495)	**0.000**	2.065 (0.635–6.719)	0.228
Tumor size (cm)	>5 vs. ≤5	3.528 (1.635–7.612)	**0.001**	4.679 (1.441–15.195)	**0.010**
Vascular invasion	Yes vs. No	2.695 (1.165–6.231)	**0.020**	0.282 (0.058–1.361)	0.115
Intra-hepatic recurrence	Yes vs. No	2.118 (0.967–4.640)	0.061	0.718 (0.192–2.689)	0.623
Extra-hepatic metastasis	Yes vs. No	2.741 (1.216–6.181)	**0.015**	2.177 (0.714–6.638)	0.171
Neo-adjuvant treatments	Yes vs. No	3.851 (1.121–13.230)	**0.032**	2.132 (0.341–13.318)	0.418
Adjuvant treatments	Yes vs. No	4.522 (1.393–14.687)	**0.012**	11.204 (2.231–56.266)	**0.003**
Liver cirrhosis	Yes vs. No	3.377 (1.258–9.062)	**0.016**	5.202 (1.324–20.435)	**0.018**
HBsAg	+ vs. -	0.542 (0.241–1.215)	0.137	0.596 (0.153–2.317)	0.455

*Abbreviation*: *HR* hazard ratio, *OS* overall survival, *CI* confidence interval

Significant data are highlighted in bold. ^a^ Correlation is significant at the 0.01 a level (2-tailed)

### TCGA data analysis of ERa transcripts and wtERa, ERb mRNA expression

**Fig. 5:**
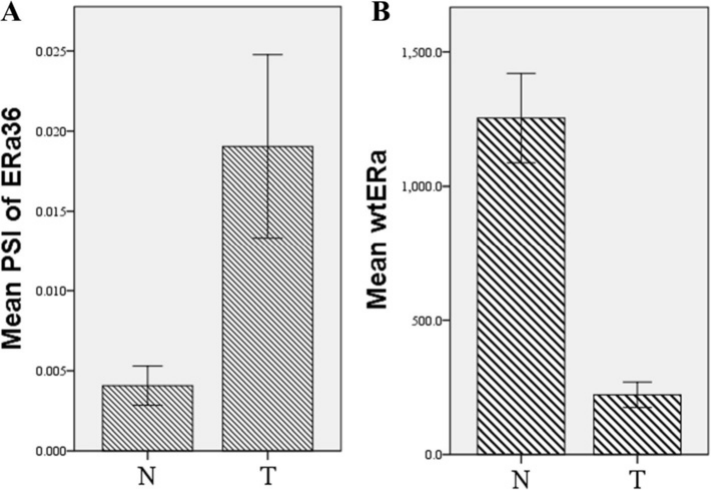
Mean Percent Spliced In (PSI) values of ERa36 and mRNA of wild type ERa in tumor (T) HCC tissues and adjacent normal (N) liver tissues. **a**. mean PSI value of ERa36 showed that the percentage of ERa36 transcript was higher in tumor tissues than in non-tumor tissues (*P* < 0.001, Error bars, 95 % CI); **b**. The mRNA expression level of wtERa was lower in tumor tissues than in non-tumor tissues (*P* < 0.001, Error bars, 95 % CI)

Because ERa36 has a different 3’ untranslated region end [11] from wtERa, wtERa and ERa36 can be discriminated by two alternate terminator (AT) events. In TCGA LIHC data set, a total of 121 HCC tumor tissues and/or 50 adjacent non-tumor tissues were included in our research for alternative splicing analysis of ERa and mRNA expression. In general, the Percent Spliced In (PSI) value of ERa36 was significant higher in HCC tissues than in adjacent non-tumor tissues (mean value 0.019 versus 0.004, *P* < 0.001, Fig. 5). In contrast, the PSI value of ERa66 was also significant higher in adjacent non-tumor tissues than in HCC tissues (mean value 0.996 versus 0.981, *P* < 0.001). Using Kendall tau rank correlation, we found that the PSI value of ERa36 was correlated with tumor status (*P* = 0.003), tumor grade (*P* = 0.002), and new tumor events (*P* =0.001). These findings support that ERa36 functions against ERa66, with the former being oncogenic but the latter being protective [13, 14], suggest that ERa36 may contribute to the development and/or progression of HCC. The high PSI value of ERa36 was not significantly correlated with risk factors, AJCC TNM & pathological stage, vascular invasion, Child-pugh classification, and age at diagnosis. Moreover, when we divided the 121 HCC patients into two groups using median PSI value as a cutoff point, we did not find significant changes in survival between two groups (log-rank *P* > 0.05).

To further reveal the relationships between PSI value of ERa36 mRNA and the levels of ERa and ERb mRNA, we found that ERa36 was significantly negatively correlated with ERa (Pearson correlation efficient = −0.403, *P* < 0.001). No significantly correlation was found between ERa36 and ERb or between wtERa and ERb. The expression of wtERa mRNA was higher in adjacent non-tumor tissues than in HCC tissues (mean value 221.54 versus 1254.00, *P* < 0.001, Fig. 5).

## Discussion

Previous reports suggest that the levels of wtERa and ERb expression were downregulated in HCC than in chronic liver disease and ER-α36 was upregulated in HCC [15, 16]. However, the relationship between different ERs and clinical features in primary or secondary HCC has not been established. In this study, we analyzed the expression patterns of wtERa, ERa36 and ERb, and studied the predictive and prognostic value of ERs in HCC using two independent cohorts and one publicly available TCGA data set. Findings from our study indicated that the mRNA expression of wtERa was negatively correlated with ERa36 transcript in patients with HCC (the TCGA data set). This finding was confirmed at protein levels analyzed by IHC of primary HCC patients from our hospital. Importantly, we have demonstrated that compared with non-tumor tissues, the expression of ERa36 is increased in primary HCC but decreased in secondary HCC, showing opposite expression patterns of ERa36 between primary HCC and secondary HCC. Furthermore, the expression of ERa36 in the primary HCC is much higher than in the secondary HCC. Therefore, the expression of ERa36 may be used to differentiate the primary HCC and the secondary one.

The estrogen pathway plays a critical role in tumorigenesis, metastasis, and response to certain therapies of HCC [4, 16, 17]. The role of wtER in HCC was investigated early in 1980s [18]. Due to multiple variants of ERa and ERb, the actual role of wtER in HCC was too complex to be defined. Several studies have reported that the expression of wtER was less in tumor tissues than in adjacent normal tissues [19, 20], which was in line with our findings on wtERa. These results indicate that wtERa may exhibit a protective role in HCC [21]. The downregulation of wtERa in HCC tumor tissues can be due to the hypermethylation of CpG sites in the promoter region of wtERa [22]. The expression of ERs can also be regulated by miRNA or lncRNA [23]. For example, the expression of wtERa in tumor tissues may also be inhibited by mir-18a, which is further controlled by tumor suppress gene P53 [24, 25]. Villa et al. reported that wtER and an exon 5-deleted ER variant could be used as classification predictors for survival of HCC [26]. The upregulation of wtERa led to the prolonged overall survival and disease free survival in primary HCC in our study.

Interestingly, the expression of novel ERa36 is higher in tumor tissues than in adjacent non-tumor tissues in our study. This finding is in line with one early report showing that that the levels of ERa36 mRNA were gradually increased from normal liver to cirrhotic liver and to HCC liver [15]. It thus appears that HCC tumor tissues are associated with the increased level of ERa36 but the decreased level of wtERa. The opposite expression of wtERa and ERa36 in HCC may suggest differential roles of ERs in HCC. It has been reported that wtERa functions as a tumor suppressor gene in some cancers including HCC [21]. Though the function of ERa36 in HCC has not yet been defined, it is known to promote the growth of other cancers such as breast cancer cells [27]. The fact that wtERa is reduced in HCC and wtERa functions as a tumor suppressor may well explained the failure of early trial of tamoxifen, as wtER antagonist, to treat HCC [6, 28]. However, recent studies have indicated that tamoxifen may inhibit HCC via ER-independent mechanisms [29].

In this study, we fail to show the association of ERa36 upregulation with survival in either primary HCC or secondary HCC from CRC. The finding is unexpected and the negative result may be due to the size of samples. The prognostic value of ERa36 has been demonstrated in some other cancers such as breast cancer [13, 28]. As a new oncogenic molecule. ERa36 may facilitate the growth, invasion and metastasis of cancers via various pathways including cancer stem/progenitor cells, and AKT survival signaling [28]. It is thus reasonable to consider it as a potential therapeutic target [29]. The finding of the increased ERa36 in HCC may suggest that HCC patients may also benefit from targeting ERa36.

## Conclusions

Using independent patient cohorts from primary/secondary HCC and TCGA database, we have determined the expression patterns of wtERa, ERa36 and ERb and their association with clinical characteristics. We have shown that the expressions of wtER and ERa36 were in opposite directions in primary HCC, and that ERa36 was increased in primary HCC tissues while decreased in secondary HCC. The high levels of wtERa mRNA appears to predict better survival of patients with HCC. The mechanism responsible for the abnormal expression of ERs in HCC remains unknown. Our current findings suggest that the expression of ERa36 protein could be a useful tool to discriminate primary HCC from secondary HCC patients from CRC, and that its oncogenic role may render it as a therapeutic target.

## Abbreviations

anti-HCV: Antibodies to the hepatitis C virus
anti-HIV: Antibodies to human immunodeficiency virus
AT: Alternate terminator
DFS: Disease free survival
ERa: Estrogen receptor alpha
ERb: Estrogen receptor beta
HCC: Hepatocellular carcinoma
mCRC: Colorectal liver metastasis
PVE: Portal vein embolization
TACE: Transcatheter arterial chemoembolization
TBS: Tris-buffered saline
TCGA: The cancer genome atlas
TNM: Tumor-node-metastasis
